# Anaphase B spindle dynamics in *Drosophila *S2 cells: Comparison with embryo spindles

**DOI:** 10.1186/1747-1028-6-8

**Published:** 2011-04-08

**Authors:** Jane de Lartigue, Ingrid Brust-Mascher, Jonathan M Scholey

**Affiliations:** 1 Department of Molecular and Cellular Biology, University of California, Davis, CA 95616, USA

## Abstract

**Background:**

In the *Drosophila melanogaster *syncytial blastoderm stage embryo anaphase B is initiated by a cell cycle switch in which the suppression of microtubule minus end depolymerization and spatial reorganization of the plus ends of outwardly sliding interpolar microtubules triggers spindle elongation. RNA interference in *Drosophila *cultured S2 cells may present a useful tool for identifying novel components of this switch, but given the diversity of spindle design, it is important to first determine the extent of conservation of the mechanism of anaphase B in the two systems.

**Results:**

The basic mechanism, involving an inverse correlation between poleward flux and spindle elongation is qualitatively similar in these systems, but quantitative differences exist. In S2 cells, poleward flux is only partially suppressed and the rate of anaphase B spindle elongation increases with the extent of suppression. Also, EB1-labelled microtubule plus ends redistribute away from the poles and towards the interpolar microtubule overlap zone, but this is less pronounced in S2 cells than in embryos. Finally, as in embryos, tubulin FRAP experiments revealed a reduction in the percentage recovery after photobleaching at regions proximal to the pole.

**Conclusions:**

The basic features of the anaphase B switch, involving the suppression of poleward flux and reorganization of growing microtubule plus ends, is conserved in these systems. Thus S2 cells may be useful for rapidly identifying novel components of this switch. The quantitative differences likely reflect the adaptation of embryonic spindles for rapid, streamlined mitoses.

## Background

Mitosis is mediated by the mitotic spindle, a cellular machine composed of microtubules (MTs) and mitotic motors [1-5]. The critical function of mitosis is revealed at its climax, during anaphase, at which point the spindle coordinates separation of the sister chromatids to opposite spindle poles (anaphase A) and spindle elongation (anaphase B) in preparation for cytokinesis [6-8]. In the *Drosophila melanogaster *syncytial blastoderm stage embryo, highly dynamic MTs drive remarkably rapid movements of the chromosomes and spindle poles. Anaphase B spindle elongation is proposed to depend on an interpolar (ip) MT sliding filament mechanism generated by homotetrameric kinesin-5 motors and an "on-off" switch orchestrated by the suppression of poleward MT flux [9-13]. The current model of the mechanism underlying this anaphase B "switch" postulates that the pre-anaphase B spindle is maintained at a steady state length by the balance between ipMT sliding and ipMT depolymerization at the poles via kinesin-13-dependent depolymerization, generating poleward flux. In response to cyclin B degradation (i) a MT catastrophe gradient causes ipMT plus ends to invade the overlap zone where outward ipMT sliding occurs; and (ii) kinesin-13 (KLP10A)-dependent depolymerization is switched off, tipping the balance of forces to allow outward ipMT sliding to push apart the spindle poles [14].

It is now recognized that the mechanisms of mitosis can vary significantly in different cell types, even within the same organism [5]. For example, in the *Drosophila melanogaster *syncytial blastoderm stage embryo multiple spindles progress rapidly and synchronously through mitosis. Cultured S2 cells, in contrast, do not contain hundreds of spindles progressing synchronously through mitosis, making them less amenable to the quantitation of spindle dynamics. However, these cells have emerged as a very useful model system for studying mitosis using RNA interference (RNAi) techniques to probe the function of candidate proteins in mitosis (and other subcellular processes) [12,15-19]. S2 cell spindles differ from those of the embryo in a number of different ways. Firstly, mitosis is much slower, about 40-50 mins from nuclear envelope breakdown (NEB) through to cytokinesis. S2 cell spindles are also less centrosome-dependent and can be formed by centrosome-independent mechanisms [20]. Despite these differences, it is possible that some of the underlying molecular characteristics of mitosis may be conserved between embryos and S2 cells, including aspects of the anaphase B switch. For example, Matos *et al. *observed a suppression of MT poleward flux at anaphase B onset [18]. In order to determine the extent of conservation of the anaphase B switch between embryos and S2 cells, and to evaluate the suitability of S2 cells for identifying novel components of the anaphase B switch, we used various S2 cell lines to examine MT dynamics in S2 cell mitotic spindles and compared the results with those obtained using embryo spindles.

## Results

### Anaphase A and B are synchronized in *Drosophila *S2 cells

**Figure 1:**
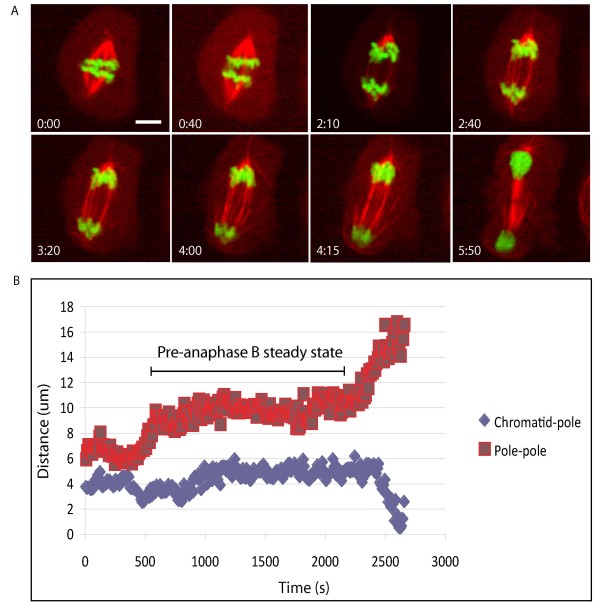
**Timing of anaphase A chromatid-to-pole movement in *Drosophila *S2 cells**. (A) Stills from a movie following anaphase A chromatid-to-pole movement in an S2 cell stably expressing histone 2B (green) and -α-tubulin (red). Time is expressed as min:sec and scale bar represents 5 μm. (B) Graph illustrating the timing of anaphase A chromatid to pole motility relative to anaphase B spindle elongation. Note that time point 0 in the graph does not correspond to that in the stills.

The *Drosophila *early embryo carries out multiple, rapid mitotic cycles characterized by 13 nuclear divisions. During cycles 10-13 the nuclei divide synchronously, without intervening cytokinesis, in a single monolayer just underneath the surface. These mitoses are generally believed to use the same mitotic machinery as in other cells, but are specifically optimized for speed [21]. Previous measurements from our lab indicate that cycle 12 spindles are an average of 10 μm in length. The length of time from NEB to telophase is approximately 350 s, with the pre-anaphase B steady state period lasting an average of 80 s (See table 1 for a summary of embryo spindle characteristics) [11]. Our measurements of S2 cell spindles illustrate that these spindles are slightly shorter at an average of 8.6 μm (± 1.7 μm) during pre-anaphase B steady state (note that the pre-anaphase B steady state refers to the period at which a steady spindle length is reached and incorporates metaphase and possibly part of prometaphase since chromosome congression was not monitored), elongating approximately 4 μm from NEB and subsequently elongating approximately 5 μm further during anaphase B (see Figure 1B for an example plot of pole-pole distance over the course of mitosis, and table 1 for a summary of S2 cell spindle characteristics). Mitosis progressed significantly more slowly, about 8 times slower, on average lasting 48 minutes (2879.6 s). Interestingly, the pre-anaphase B steady state comprised a much more substantial portion of the total length of mitosis (approximately 70% vs 20% of the total length in embryos), with an average duration of approximately 35 min (2126.2 s) (Table 1). This could reflect the fact that cell cycle checkpoints are generally believed to be less stringent in the embryo or that chromosome congression takes longer in S2 cells.

**Table 1:** Summary of comparative data

	***Drosophila *syncytial embryo (cycle 12)**^**10,11**^	*Drosophila *S2 cells
**Duration:**		
**NEB-telophase**	5-6 min	40-50 min
**Pre-anaphase B steady** **state**	1-2 min	30-40 min

**Pre-anaphase B spindle** **length**	10 μm	8.6 μm
**Anaphase B spindle length** **change**	5 μm	5 μm

**MT flux rates:**		
**Pre-anaphase B**	0.053 ± 0.013 μm/s	0.05 ± 0.006 μm/s
**Anaphase B**	0.008 ± 0.019 μm/s	0.03 ± 0.007 μm/s

**Anaphase B rate**	0.08 ± 0.015 μm/s	0.017 μm/s

**Chromosome segregation** **rate**	0.11 ± 0.019 μm/s	0.0098 ± 0.0047 μm/s

**MT plus end distribution** **at anaphase B**	Redistribution to the equator	Partial redistribution to the equator

**MT turnover at pole:**		
**Pre-anaphase B**	81.0 ± 14.3%	113.1 ± 12.3%
**Anaphase B**	45.8 ± 11.2%	71.8 ± 21.2%

In the embryo anaphase A chromosome segregation proceeds at an average rate of 0.1 μm/s during cycle 12 (Table 1) and comes almost to completion prior to the initiation of anaphase B spindle elongation [10]. In other organisms anaphase A and B appear to be much more synchronized and previous reports have indicated that this may also be the case in *Drosophila *S2 cells [18,22]. In order to investigate the timing and rate of anaphase A and B in S2 cells, we used a cell line in which the chromosomes were labelled by the stable expression of GFP-tagged histone 2B, in addition to the stable expression of RFP-α-tubulin to label the spindle. Using time-lapse microscopy we imaged concanavalin A-flattened S2 cells from NEB through to cytokinesis (Figure 1A). In the majority of cases (60%) we found that chromosome to pole motility occurred at the same time as anaphase B spindle elongation in these cells. In the remaining cells chromosome to pole motility began prior to spindle elongation in the same manner as the syncytial embryo. Chromosome to pole motility occurred at an average rate of 0.0098 μm/s ± 0.0047 μm/s (Figure 1B; n = 10; table 1), approximately 10 times more slowly than in the embryo.

### The suppression of MT poleward flux at anaphase B onset is linked to spindle elongation during anaphase B

**Figure 2:**
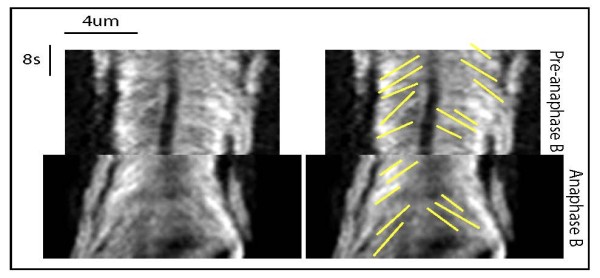
**Fluorescent tubulin speckle trajectories pre-anaphase B and during anaphase B spindle elongation**. Kymographs generated from fluorescent speckle time lapse movies of S2 cells expressing low levels of GFP-α-tubulin during pre-anaphase B and anaphase B, illustrating tubulin speckle trajectories (yellow lines).

During the pre-anaphase B stage in many animal and plant spindles there is a characteristic continuous poleward translocation of MT subunits, termed "flux". In the *Drosophila *syncytial embryo (cycle 12) experiments performed in our lab indicate that MT flux progresses at a rate of 0.053 μm/s on average pre-anaphase B and then is switched off at the onset of anaphase B (rate = 0.008 μm/s; table 1), most likely due to inactivation of KLP10A-mediated minus-end depolymerisation at the poles [9,13,21]. It has previously been reported that MT flux may also be attenuated in *Drosophila *S2 cells [18]. To further investigate the relationship between poleward flux and anaphase B spindle elongation we used an S2 cell line expressing GFP-α-tubulin under the control of a leaky metallothionein promoter. These cells express a low level of GFP-α-tubulin such that the spindle is not uniformly labelled and individual tubulin "speckles" can be observed. We tracked the movement of these tubulin speckles using kymography and determined that the pre-anaphase B MT poleward flux rate was 0.05 μm/s, similar to the rate of poleward flux previously observed in the embryo. Following onset of anaphase B the flux rate was attenuated to 0.03 μm/s (Figure 2; Table 2; *P < 0.0001*) but not completely suppressed as in the syncytial embryo. We also measured the flux rate using fluorescence recovery after photobleaching (FRAP). We photobleached a 1 μm region in the half spindle and followed its poleward movement over time using line scans. Using these measurements we also observed an attenuation of the MT poleward flux rate after anaphase B onset (data not shown).

**Table 2:** Partial suppression of microtubule poleward flux at anaphase B onset

	Pre-anaphase B (μm/s)	Anaphase B (μm/s)
**Average MT flux rate** (± standard deviation)	0.05 ± 0.006	0.032 ± 0.007

**N**	9 spindles	10 spindles
	85 speckles	48 speckles

Average rate of poleward microtubule flux as measured by fluorescence speckle microscopy, measure in μm/s, mean ± standard deviation.

**Figure 3:**
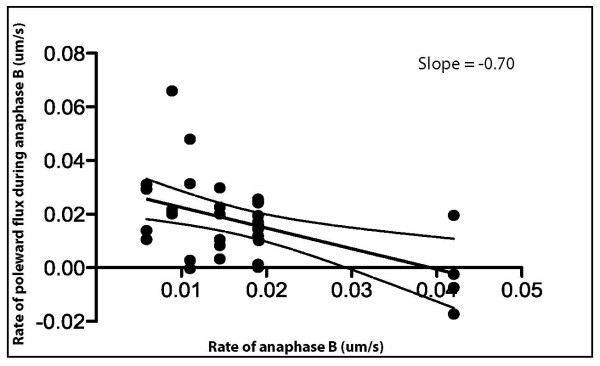
**The suppression of microtubule poleward flux is linked to spindle elongation during anaphase B**. Graph illustrating the linear inverse relationship between poleward flux during anaphase B and the rate of anaphase B. Data points display the behaviour of individual fluorescent tubulin speckles within individual spindles of wild-type S2 cells expressing low levels of GFP-α-tubulin. The 95% confidence intervals for the line of best fit are also shown.

During anaphase B in the syncytial embryo the spindle elongates at a linear rate of 0.08 μm/s on average (Table 1), whilst speckles move away from the equator at the same rate as the poles, consistent with ipMT sliding. It has been shown that the suppression of flux couples ipMT sliding to spindle elongation, thereby controlling the onset and rate of anaphase B [9]. In order to determine whether the partial suppression of flux in S2 cells has a similar role, we plotted the rate of poleward MT flux against the rate of anaphase B. The rate of anaphase B spindle elongation is much slower in S2 cells, proceeding on average at 0.017 μm/s (Table 1). As in the embryo, we observed an inverse linear relationship between flux and pole-pole separation, such that low flux rates correlated with high anaphase B rates (Figure 3), indicating that a similar mechanism of anaphase B regulation occurs in S2 cells. In the syncytial embryo, the relationship between flux and sliding is revealed through the use of antibody and dominant negative protein inhibitors of the chromokinesin KLP3A (slope = -0.54) [9]. In S2 cells this relationship was revealed in wildtype cells with a steeper slope of -0.70 (Figure 3).

### MT plus ends redistribute at anaphase B onset

**Figure 4:**
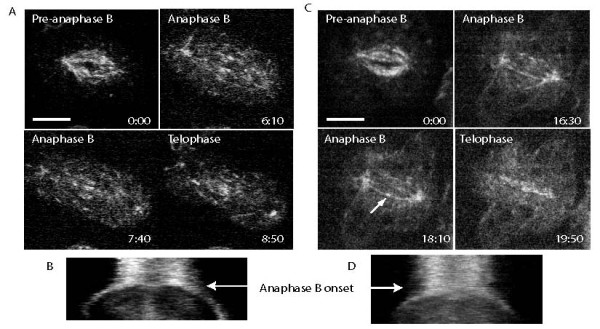
**Microtubule plus ends redistribute at anaphase B onset**. (A and C) Distribution of the microtubule tip tracker EB1 over time in S2 cells stably expressing RFP-EB1. Time is shown in min:sec and scale bars represent 5 μm. Two different phenotypes were observed. Arrow in bottom left panel of C indicates puncta of EB1 fluorescence visible at the equator of individual MT bundles. Tight redistribution of the plus ends (A) to the equator was seen in 25% of cells. (B and D) Whole spindle kymographs demonstrating EB-1 distribution across the spindle over time.

Our current model of anaphase B in the *Drosophila *syncytial embryo posits that a mechanical switch, composed of suppression of minus-end depolymerization at the poles combined with redistribution of ipMT plus ends to the overlap region at the spindle equator, initiates anaphase B spindle elongation (Table 1) [14]. We have already established that suppression of flux is conserved between embryos and S2 cells. Using an S2 cell line stably expressing an RFP tagged form of the MT plus end marker EB1, we were able to examine the distribution of MT plus ends across the spindle over time, to determine if there is a corresponding redistribution to the overlap zone in S2 cells. We generated whole spindle kymographs of EB1-labelled spindles from time-lapse videos (Figure 4A-D). We observed a redistribution of EB1 and a general reduction in overall fluorescence across the spindle. However, unlike in the embryo, the EB1 fluorescence did not concentrate so tightly at the spindle equator in the ipMT overlap region in every cell (Figure 4C and 4D). Indeed, only 25% of the cells observed demonstrated this phenotype (Figure 4A and 4B). In the remaining cells, puncta of EB1 fluorescence can be seen at the equator of individual MT bundles (e.g. Figure 4C bottom left) but EB1 was also seen to be dispersed throughout the half-spindles during anaphase B.

### Anaphase B spindles exhibit a difference in MT turnover

**Figure 5:**
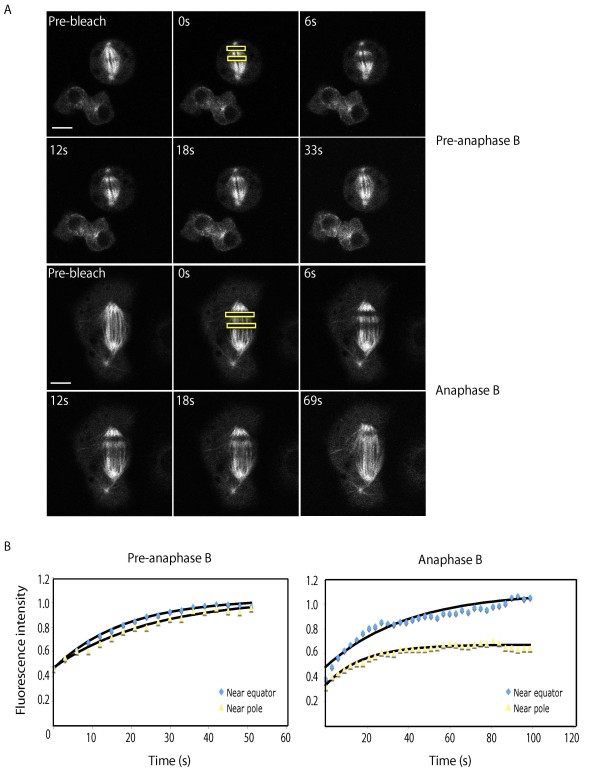
**Anaphase B spindles exhibit a spatial difference in microtubule turnover**. (A) Micrographs of a pre-anaphase B and anaphase B spindle bleached simultaneously at two separate regions proximal to either the equator or pole (yellow boxes). (B) Normalized fluorescence recovery curves of the two bleached zones and exponential fits (black line). Scale bar represents 5 μm.

Another contributing factor to the formulation of our anaphase B switch model in the syncytial embryo was the observation that there is a spatially regulated change in MT dynamics at anaphase B onset. FRAP studies performed in our lab indicated that although the half-time of recovery of spindle MTs was the same prior to and during anaphase B in the wild-type embryo, there was a notable position-dependent difference in the percentage of recovery during anaphase B, such that regions proximal to the spindle pole exhibited a substantial reduction in recovery (45.8% at the pole vs 85.8% at the equator; Table 1) [14]. By performing the same FRAP experiments in S2 cells we observed that this reduction in recovery is conserved in these spindles. After bleaching regions proximal to the spindle pole and equator in pre-anaphase B and anaphase B spindles (Figure 5A) and plotting normalized fluorescence recovery curves of the two bleached zones and exponential fits (Figure 5B), we determined that percentage recovery at the pole reduced from 113.9% pre-anaphase B to 78.8% during anaphase B (Table 3). The half time of recovery was much slower and more variable in S2 cells in comparison to the embryo, ranging from 5.2 to 18.4 s, compared to values ranging from 2.8 to 10 s in the embryo.

**Table 3:** Microtubule turnover during pre-anaphase B and anaphase B

	Pre-anaphase B			Anaphase B	
	**t1/2**	**% recovery**		**t1/2**	**% recovery**

**Pole**	16.6 ± 2.8	98.5 ± 12.2	**Pole**	16.8 ± 5.14	71.8 ± 21.2

**Equator**	10.7 ± 2.2	107.5 ± 11.4	**Equator**	17.5 ± 8.72	113.1 ± 12.3

**N**	8	8	**N**	8	8

t1/2 and percentage recovery values following FRAP at regions proximal to the pole and the equator in normalized spindles during pre-anaphase B and anaphase B. Values are mean ± standard deviation.

## Discussion

The *Drosophila *syncytial blastoderm stage embryo is an essential tool in the study of mitosis. Injecting antibody and dominant negative protein inhibitors into the embryo, often in combination with the use of mitotic mutants permits detailed study of the role of mitotic proteins [21,23]. However, the generation and characterization of reagents used in these embryo studies is often difficult and time consuming and it would be extremely useful to be able to rapidly identify potential novel components of the anaphase B switch prior to making this investment. *Drosophila *S2 cells, though less amenable to quantitation of spindle dynamics, are very useful for RNAi-mediated depletion of mitotic proteins. Indeed, they have previously been used to probe the functions of several mitotic proteins in S2 cell spindle assembly [12,15,16,24,25]. In order to assess the utility of S2 cell RNAi in identifying novel components of the anaphase B switch, it is important to understand whether this switch and the molecular mechanisms underlying it are conserved between embryos and S2 cells.

There is variation between and even among different species in the timing of anaphase B spindle elongation relative to anaphase A chromosome segregation [21]. Spindles of the syncytial embryo undergo a distinct anaphase A chromosome segregation whilst maintaining a constant spindle length, which is followed just prior to anaphase A completion, by anaphase B spindle elongation. In S2 cells, however, anaphase A and B are more synchronized, with chromosome segregation occurring just prior to or at the same time as spindle elongation and completing at approximately the same time. Chromosome to pole motility also occurs at a much slower rate in S2 cells than in the embryo, at an average of 0.0098 μm/s (Table 1).

A key feature of the switch from constant spindle length pre-anaphase B to spindle elongation during anaphase B in the embryo is the suppression of poleward microtubule flux. Our data confirm and extend previous findings that this component of the anaphase B switch is conserved in S2 cells [18] and we further observe that there is an inverse correlation between the rate of poleward flux and the rate of spindle elongation, as in embryos. However, while flux is normally completely suppressed in embryonic spindles, it is only partially suppressed in S2 cell spindles (Table 2) and to a variable extent (Figure 3). In embryos, mitotic spindles are adapted to carry out the multiple rapid and synchronous mitoses that occur as the fertilized egg develops into the multicellular larva - here the complete suppression of poleward flux allows the spindle to elongate at its maximal rate. In S2 cells there is no such requirement for rapid, synchronous divisions, and here, the incomplete suppression of flux is consistent with a more stately, less streamlined pace of progression through mitosis.

An additional factor influencing this difference may be the contribution of poleward flux to chromosome segregation in S2 cells, which continues throughout spindle elongation. There is some debate in the literature as to the role of flux in chromosome segregation in S2 cells. Buster *et al. *reported that MT minus-end depolymerization associated with flux was the main driving force behind chromosome to pole motility [26]. However, a subsequent paper by Matos *et al. *argued that Pacman activity, namely kinetochore motility coupled to MT plus end depolymerization, drives anaphase A in S2 cells, since the reduction in flux at anaphase B onset does not affect the mean velocity of kinetochore (k) MT shortening [18]. The inverse linear relationship we observed between the rates of poleward flux and anaphase B indicates that suppression of flux is linked to spindle elongation at anaphase B onset. It is possible that flux is partially suppressed at anaphase B onset in S2 cells in order to permit spindle elongation, but that the lower rate of MT flux in combination with Pacman mechanisms continues to drive chromosome to pole motility.

The second component to the proposed anaphase B switch in *Drosophila *embryos is the redistribution of ipMT plus ends to the overlap zone at the spindle equator. We examined the distribution of the MT plus end tracking protein EB1 to determine if this phenomenon was conserved in S2 cells. While there was a difference in the EB1 distribution at anaphase B onset, only 25% of cells displayed concentrated EB1 fluorescence at the overlap region as in the embryo. In the majority of cells there was a general reduction in EB1 fluorescence across the spindle and redistribution away from the poles and towards the equator, but fluorescence was not as tightly redistributed to the midzone as in the embryo (Figure 4). A tight distribution of MT plus ends at the overlap region in S2 cells may not be necessary, since such a rapid and coordinated spindle elongation is not needed. It is also possible that EB1 binds to the polymerizing kMT ends on segregating sister chromatids [27], which would be moving away from the spindle midzone and therefore make the EB1 distribution appear more broadly spread across the spindle in S2 cells. Indeed, EB1 fluorescence becomes progressively more concentrated at the midzone over the course of anaphase B, by which point the chromatids should have fully separated (Figure 4B).

We also examined MT dynamics at regions proximal to the pole and the equator using FRAP and, as observed in the embryo, found that there was a reduction in fluorescence recovery close to the pole (Figure 5; Table 3). This further suggests that although the redistribution of EB1 fluorescence to the spindle midzone is not so pronounced in S2 cells, the alteration in spindle MT dynamics at anaphase B onset is conserved, such that MTs proximal to the pole stabilize. In future work it would be interesting to determine whether the role of cyclin B degradation in these changes is also conserved and to model the S2 cell data in a similar manner to the embryo. This would allow us to model the potential outcomes of RNAi depletion of candidate proteins involved in the anaphase B switch and interpret further experimental data.

There is a substantial level of conservation in the basic mechanisms underlying anaphase B in both the *Drosophila *syncytial blastoderm stage embryo and cultured S2 cells. Therefore, it would seem that RNAi studies might provide a useful means of identifying novel components of the anaphase B switch. However, there are a number of features of S2 cells that we feel may compromise their usefulness for such studies. Firstly, it is much more difficult to achieve the same level of quantitation of spindle dynamics in S2 cells as in the embryo since the mitotic index is much lower, with only 2-3% of cells undergoing mitosis at any one time. Furthermore, mitosis is not synchronized in S2 cells and, although there have been reports of methods to synchronize S2 cells and increase their mitotic index, we were unable to successfully achieve this goal over the course of our study. Since anaphase A and B occur much more synchronously than in the embryo, it is therefore important to remember that kMTs will still be present along with ipMTs in the S2 cell spindle during anaphase B and may contribute to any measurements of MT dynamics that are undertaken - because of the temporal separation of anaphase A and B in embryos, it is possible to specifically focus on ipMTs. In addition, S2 cells require flattening to the coverslip or culture dish prior to imaging. Concanavalin A represents the optimal means of achieving flattened cells, however it is important to minimise the amount of time that cells are imaged after flattening since it can begin to interfere with cytokinesis. We found that the optimal imaging time was from 20 minutes up to 2 hours after flattening (data not shown). Furthermore, S2 cells are very sensitive to light and prolonged exposure to the imaging laser can damage the cells and arrest mitosis. We therefore only imaged cells that had entered anaphase less than 40 minutes after NEB, and added Vitamin C (ascorbic acid) to the medium to act as an antioxidant and avoid build up of cell damage. Finally, compared to untransformed cells in other wild-type organisms, mitosis in the S2 cell line is more variable and often looks abnormal even in untreated cells. It is therefore of great importance to establish robust control samples and to perform objective imaging and image analysis in order to accurately discriminate *bona fide *RNAi-induced effects from simple cellular variation.

## Conclusions

In conclusion, we have demonstrated that the basic components of an anaphase B switch previously observed in the *Drosophila *syncytial embryo are conserved in cultured S2 cells, at least qualitatively. Thus S2 cells may provide a useful model for identifying novel molecular components of the anaphase B switch via RNAi, bearing in mind that a number of technical difficulties peculiar to the study of anaphase B in this cell line will need to be surmounted first. The quantitative differences, which can be generalized to an "all-or-none" versus a "partial" switch in embryo versus S2 cell spindles, respectively, are likely to reflect differences in the speed and synchronicity of mitosis and the structure of the spindles in the two systems.

## Methods

### Cell culture

All experiments reported here were conducted on *Drosophila *S2 cells cultured in 1× Schneider's *Dosophila *medium (Gibco) supplemented with 10% FBS, 1 unit/ml penicillin, and 1 μg/ml streptomycin, in 75 cm^2 ^flasks at 27°C. Prior to microscopy, S2 cells were flattened onto glass-bottomed culture dishes (MatTek Corporation) coated with 0.25 mg/ml concanavalin A (Sigma Aldrich) for 20 minutes. Vitamin C was also added to the medium to act as an antioxidant and minimise cell death during imaging.

### Time lapse microscopy

Time-lapse images were collected using an Olympus microscope equipped with an UltraView spinning disk confocal head (Perkin Elmer) with a 100× 1.35 NA objective. A single confocal plane was acquired at a rate of 5-10 s/frame at 24-26°C and recorded using a charge-coupled device camera (Orca II; Hamamatsu Photonics).

### Fluorescent speckle microscopy and kymography

To measure flux rates we used S2 cells stably expressing GFP-α-tubulin under the control of a leaky inducible metallothionein promoter (a gift from Prof. Helder Maiato, University of Porto, Portugal), which express low levels of GFP-α-tubulin without induction. Images were analyzed using MetaMorph Imaging Software (Universal Imaging). The Sharpen and Low-Pass Filter commands were applied and speckle movement was quantified using kymography analysis, in which moving speckles appeared as oblique lines whose slope corresponds to their rate of movement. All calculations were performed in Microsoft Excel and flux rate was calculated relative to the movement of the spindle poles over time, such that the movement of the pole was subtracted from that of the speckle.

### Fluorescence recovery after photobleaching

For FRAP experiments we used S2 cells stably expressing GFP-α-tubulin (a gift from Dr. David Sharp) and a laser-scanning confocal microscope (FV1000; Olympus) with a 60× 1.40 NA objective. Image acquisition was performed using the Fluoview software (version 1.5; Olympus). Cells were imaged using the 488 nm line from an argon laser, whilst a separate 405 nm laser was used to photobleach GFP-α-tubulin, allowing simultaneous imaging and bleaching. The spindle was bleached in rectangles of 1 μm diameter proximal to either the pole or equator and images were acquired every 3 s. The spindles were corrected for movement using Matlab (Mathworks) and the fluorescence intensity of the bleached regions over time were measured using MetaMorph imaging software. All data were normalized by I(t) = [F(t)T_pre_]/[T(t)F_pre_], where F(t) and T(t) are the mean fluorescence in the FRAP region and in the entire spindle at time t, and F_pre _and T_pre _are the mean fluorescence in the FRAP region and in the entire spindle just before the bleach. The percentage recovery was obtained by using the calculation (F_inf_-F_0_)/(F_pre_-F_0_), where F_0 _is the mean intensity in the FRAP region just after photobleaching and F_inf _is the final fluorescence obtained from the single exponential fit. The fit to the data also yielded the recovery half time.

### EB-1 distribution

The distribution of EB1 across the spindle over time was analyzed using S2 cells stably expressing RFP-EB1 (also a gift from Dr Steve Rogers). A time lapse movie was acquired as described above, the spindle was aligned using Matlab and then a whole spindle kymograph was generated using MetaMorph imaging software.

### Chromosome to pole motility

Chromosome-to-pole motility rates were measured using S2 cells stably expressing GFP-histone2B and RFP-α-tubulin (a gift from Prof. Ronald D. Vale, University of California, San Francisco, USA).

## Competing interests

The authors declare that they have no competing interests.

## Authors' contributions

JL carried out experiments and statistical and other data analyses and drafted the manuscript. JMS is the PI of the laboratory; he and IBM conceived of the study and participated in its design, and helped to draft/edit the manuscript. All authors read and approved the final manuscript.
